# Computationally efficient permutation-based confidence interval estimation for tail-area FDR

**DOI:** 10.3389/fgene.2013.00179

**Published:** 2013-09-17

**Authors:** Joshua Millstein, Dmitri Volfson

**Affiliations:** ^1^Division of Biostatistics, Department of Preventive Medicine, Keck School of Medicine, University of Southern CaliforniaLos Angeles, CA, USA; ^2^Pfizer, Neuroscience Research UnitCambridge, MA, USA

**Keywords:** false discovery rates, multiple testing, simultaneous inference, gene expression, sleep

## Abstract

Challenges of satisfying parametric assumptions in genomic settings with thousands or millions of tests have led investigators to combine powerful False Discovery Rate (FDR) approaches with computationally expensive but exact permutation testing. We describe a computationally efficient permutation-based approach that includes a tractable estimator of the proportion of true null hypotheses, the variance of the log of tail-area FDR, and a confidence interval (CI) estimator, which accounts for the number of permutations conducted and dependencies between tests. The CI estimator applies a binomial distribution and an overdispersion parameter to counts of positive tests. The approach is general with regards to the distribution of the test statistic, it performs favorably in comparison to other approaches, and reliable FDR estimates are demonstrated with as few as 10 permutations. An application of this approach to relate sleep patterns to gene expression patterns in mouse hypothalamus yielded a set of 11 transcripts associated with 24 h REM sleep [FDR = 0.15 (0.08, 0.26)]. Two of the corresponding genes, Sfrp1 and Sfrp4, are involved in wnt signaling and several others, Irf7, Ifit1, Iigp2, and Ifih1, have links to interferon signaling. These genes would have been overlooked had a typical a priori FDR threshold such as 0.05 or 0.1 been applied. The CI provides the flexibility for choosing a significance threshold based on tolerance for false discoveries and precision of the FDR estimate. That is, it frees the investigator to use a more data-driven approach to define significance, such as the minimum estimated FDR, an option that is especially useful for weak effects, often observed in studies of complex diseases.

## Introduction

False Discovery Rates (FDR) have become a widely used multiple testing strategy that is much less conservative than family-wise error rate (FWER) methods such as the Bonferroni and Šidák corrections when multiple null hypotheses are false (Benjamini and Hochberg, 1995; Yekutieli and Benjamini, 1999; Efron and Tibshirani, 2002; Farcomeni, 2008). Storey and Tibshirani (2003; Storey, 2002) proposed an approach (denoted below as ST) in which FDR is *estimated* for a fixed rejection region, in contrast to the more traditional approach in which FDR is *controlled* that is, the error rate is fixed and the rejection region is estimated. Their approach incorporates an estimator of the proportion of true null hypotheses, π_0_, which increases power over the original Benjamini and Hochberg (1995) method when a substantial proportion of null hypotheses are false.

Permutation-based testing approaches are especially important in genomic studies because severe multiple testing conditions require parametric tests to rely exclusively on the extreme tails of the distribution, which are notoriously inaccurate models of real data. Parametric FDR methods can be implemented as non-parametric permutation-based approaches by computing empirically approximated *p*-values in a preliminary step (Yekutieli and Benjamini, 1999; Storey and Tibshirani, 2003; Yang and Churchill, 2007; Efron, 2010b) assuming exchangeability across tests under the null (Efron, 2007b). Ironically, it is often difficult to apply permutation approaches in ultra-high dimensional testing settings where they would seem to be most useful due to their intensive computational requirements. In view of this limitation, it is clearly important to address the question of the precision of the FDR estimate when just a small number of permutations have been conducted, and more generally, how precision depends on the number of permutations.

Also, the framing of FDR as an underlying quantity that can be estimated naturally leads to the question of the precision of the estimate. In the case of the ST and similar estimators, there is no explicit control of the FWER inherent in the estimate (Ge et al., 2003), and unlike a *p*-value, the magnitude of the estimate does not directly reflect the probability that the observed results are due to chance alone. It is therefore of paramount importance to know the precision of the FDR estimate. However, despite interest in quantifying uncertainty in the FDR estimate (Yekutieli and Benjamini, 1999; Storey, 2002; Owen, 2005; Efron, 2007b, 2010a; Schwartzman, 2008; Schwartzman and Lin, 2011), none of this work has resulted in a practical permutation-based CI estimator for FDR under large-scale testing conditions where there are dependencies between tests.

We propose a permutation-based tail-area FDR estimator that incorporates a novel tractable estimator of π_0_, which is a simple function of counts of observed and permuted test outcomes. The development of a novel FDR CI estimator is then achieved by leveraging the tractability of the proposed point estimator, treating positive test counts as binomial random variables, and including a novel overdispersion parameter to account for dependencies among tests. Because the CI estimator explicitly incorporates the number of permutations conducted, indirect guidance is provided regarding whether that number is sufficient.

Evidence has been found in mice linking DNA variation to variation in 24 h REM sleep, possibly mediated by chronic differences in gene expression (Winrow et al., 2009; Millstein et al., 2011). Here we report an application of the method to identify gene expression features in the hypothalamus associated with variation in 24 h REM sleep in a segregating population of mice. Not only is FDR estimated and uncertainty quantified using the proposed approach, but a significance threshold is also selected a posteriori, in a data-driven manner.

## FDR estimators

### Permutation-based FDR point estimator

Positive FDR is the expected proportion of tests called significant that are actually true null hypotheses given that the number of significant tests is greater than zero,

(1) $$\text{FDR}=E\left[\frac{F}{S}|S>0\right]=E\left[\frac{S-T}{S}|S>0\right]$$

Table 1 provides a two-by-two table summary of possible test outcomes, where *m* denotes the total number of tests conducted, *m*_0_ and *m*_1_ the number of true and false null hypotheses, respectively, *S* the total number of tests called significant, *F* the number of rejected null hypotheses that are true (false discoveries), and *T* the number of rejected null hypotheses that are false (true discoveries). The goal is to estimate FDR for a fixed significance threshold, thus *S*, *F*, and *T* depend on that threshold. The null distribution for a test statistic can often be approximated using a permutation procedure where the data are permuted repeatedly, with a set of test statistics generated for each replicate permuted dataset. Permuted test results will be identified here with a ^*^ and a subscript, e.g., S^*^_i_ denotes the count of positive tests for the *i*th permuted dataset of *B* permutations. By design there are no false null hypotheses for tests of permuted data, consequently,

(2) $$\frac{E[F_{i}^{*}]}{m}=\frac{E[F_{i}^{*}]}{m_{0}^{*}}$$

**Table 1:** **Hypothesis test outcomes**.

	**Called significant**	**Called not significant**	
Null true	*F*	*m*_0_ – *F*	*m*_0_
Null false	*T*	*m*_1_ – *T*	*m*_1_
	*S*	*m* – *S*	*m*

The principal assumption underlying most permutation testing approaches is exchangeability of observations under the null hypothesis, implying that the expected proportion of positive tests among true null hypotheses is the same in observed and permuted results that is,

(3) $$\frac{E[F_{i}^{*}]}{m_{0}^{*}}=\frac{E[F]}{m_{0}}$$

By the properties of Table 1 we can express the expected proportion of observed false positives among true null hypotheses as,

(4) $$\frac{E[F]}{m_{0}}=\frac{E[S]-E[T]}{m-E[T]-(m_{1}-E[T])}\approx \frac{E[S]-E[T]}{m-E[T]}$$

which introduces the term, (*m*_1_ − *E*[*T*]), corresponding to the lower right cell of Table 1, the number of false null hypotheses called not significant. To facilitate the construction of a tractable estimator, we use the approximation that *m*_1_ − *E*[*T*] = 0. Below, we show in simulated data and provide additional arguments that this approach yields a conservative estimator relative to the ST approach yet anti-conservative relative to Benjamini and Hochberg (1995), and moreover, when *m*_0_/*m* is close to one, the bias is extremely small.

Rearranging Equation 4, we can generate an expression for *E*[*T*] as,

(5) $$E[T]=\frac{mE[F]/m_{0}-E[S]}{E[F]/m_{0}-1}.$$

In results from permuted data, by design, m^*^_1_ = 0 ⇒ T^*^ = 0, m^*^_0_ = *m*, and F^*^_i_ = S^*^_i_. Thus, we can express the expected number of false null hypotheses called significant as,

(6) $$E[T]=\frac{E[S]-E\left[S_{i}^{*}\right]}{1-E\left[S_{i}^{*}\right]/m}.$$

Storey and Tibshirani (2003) (see Remark A) noted that *E*[*F/S*] ≈ *E*[*F*]/*E*[*S*] when *m* is large, where the right hand expression has been described as the “marginal” FDR (mFDR; Tsai et al., 2003; Storey et al., 2007). We derive the following point estimator by using the mFDR expression, the fact that *E*[*F*] = *E*[*S*] − *E*[*T*], Equation 6, substituting *S* as an estimator for *E*[*S*], and substituting ${\bar{S}}^{*}$ for *E*[S^*^_i_], yielding the elegant expression,

(7) $$\text{F}\hat{\text{D}}\text{R}=\frac{{\bar{S}}^{*}}{S}\frac{1-S/m}{1-{\bar{S}}^{*}/m}.$$

Equation 7, can be related to the framework described by Storey and Tibshirani (2003) for a permutation-based FDR estimator. Their approach was chiefly described for a set of test results in the form of *p*-values, but they also proposed a permutation testing implementation that involved empirically adjusting the *p*-values using results from the permuted data prior to application of the proposed method. By rewriting their expression in terms of observed and permuted test results, $\text{F}\hat{\text{D}}\text{R}={\hat{\pi }}_{0}{\bar{S}}^{*}/S$, where ${\hat{\pi }}_{0}$ is the estimator of the proportion of true null hypotheses, *m*_0_/*m*. Equation 7 can be related to this framework by describing the factor on the far right as an estimator of the proportion of true null hypotheses that is,

(8) $${\hat{\pi }}_{0}=\frac{1-S/m}{1-{\bar{S}}^{*}/m}.$$

A relation can also be described between the estimator of 8 and ${\hat{\pi }}_{0}$ proposed by Storey (2002),

(9) $${\hat{\pi }}_{0}=\frac{\#\{p_{i}>\lambda \}}{(1-\lambda )m},$$

where *p*_*i*_ is a *p*-value for the *i*th test and λ is a tuning parameter often chosen by a smoothing algorithm (Storey and Tibshirani, 2003). A similar formula and heuristic parameter for determining ${\hat{\pi }}_{0}$ were also proposed by Efron (2010b). The expressions in 8 and 9 are equivalent if λ, bounded by 0 and 1, is fixed at the empirically adjusted *p*-value significance threshold. An important advantage of fixing lambda as proposed is that the assumption of a uniform *p*-value distribution under the global null is not required, unlike the ST approach. Storey (2004) showed that for the estimator in 9, $E[{\hat{\pi }}_{0}]\;>\;\pi_{0}$ when *p*-values corresponding to true null hypotheses are uniformly distributed and $E[\text{F}\hat{\text{D}}\text{R}]=\text{FDR}$, a potentially conservative bias. The bias occurs if there are false null hypotheses with *p*-values greater than λ and this bias tends to increase as λ decreases, though the variance of ${\hat{\pi }}_{0}$ decreases as λ decreases (Storey, 2004). Efron (2010b) proposed the equivalent of fixing λ = 0.5. The ST smoothing algorithm also results in a choice of λ substantially greater than the significance threshold, therefore the ${\hat{\pi }}_{0}$ and consequently $\text{F}\hat{\text{D}}\text{R}$ proposed here are more conservative yet with smaller variance than those proposed by Storey and Tibshirani (2003). However, the FDR estimator proposed here is less conservative than the Benjamini and Hochberg (1995) approach, which implicitly assumes ${\hat{\pi }}_{0}=1$ (Storey and Tibshirani, 2003). We show in Appendix A that the proposed estimator, ${\hat{\pi }}_{0}$, is consistent in *n* and *m*.

### FDR confidence interval estimator

The variance of $\text{F}\hat{\text{D}}\text{R}$ depends not only on its magnitude but also on other factors such as the number of positive tests. Unlike a *p*-value, the magnitude of $\text{F}\hat{\text{D}}\text{R}$ does not necessarily correspond closely to the likelihood that an observed result, i.e., an observation of $\text{F}\hat{\text{D}}\text{R}$ that is less than one, is due to chance alone, and the CI estimate can be informative in this way. The FDR CI estimator is especially useful when there is substantial uncertainty in the precision of the point estimate. For instance, suppose hypothetically that a specific high-throughput experiment yielded a minimum $\text{F}\hat{\text{D}}\text{R}=0.5$, corresponding to a set of 100 potential gene targets. It is possible that the observed value is due to chance alone (no false null hypotheses), however, if it is known that the FDR estimate is reasonably precise and follow-up validation experiments are not prohibitively expensive, then despite the high FDR these results could be quite valuable, implying that ~50 of the 100 tests are true discoveries (false null hypotheses). The CI estimator could be used to distinguish between the two scenarios, potentially salvaging useful results from a study that might otherwise be dismissed as not significant. That is, an investigator may occasionally be willing to tolerate a relatively large proportion of false discoveries if the estimated proportion of true discoveries is known to be reasonably precise.

The closed-form structure of $\text{F}\hat{\text{D}}\text{R}$ (Equation 7) permits the development of a CI estimator by treating positive test counts as binomial random variables (Appendix B) and applying the delta method after a log transformation (Appendix C). The resulting estimator has the simple form,

(10) $$\begin{array}{l} \text{Var}\left[\log\left(\text{F}\hat{\text{D}}\text{R}\right)\right]=\sigma_{\text{FDR}}^{2}=\frac{m}{\left(\sum_{i}S_{i}^{*}\right)\left(m-{\bar{S}}^{*}\right)}+\frac{m}{S\left(m-S\right)}\\ \text{or equivalently},\;\sigma_{\text{FDR}}^{2}=\frac{1}{\left(\sum_{i}S_{i}^{*}\right)}+\frac{1}{mB-\sum_{i}S_{i}^{*}}+\frac{1}{S}+\frac{1}{m-S}. \end{array}$$

The expression for $\text{F}\hat{\text{D}}\text{R}$ in 7 can be recognized as having the simple form of an odds ratio between the observed and permuted test results (Appendix C), and the second form of the expression for the variance in 10 can likewise be recognized as analogous to the well-known variance estimator for the log odds ratio (Woolf, 1955). Interestingly, under conditions that will often hold in large-scale testing paradigms, a small number of positive tests relative to the total number of tests, expression 10 simplifies to,

(11) $$\underset{\frac{m}{m\;-\;{\bar{S}}^{*}}\to 1,\;\frac{m}{m\;-\;S}\to 1}{\lim}\sigma_{\text{FDR}}^{2}=\frac{1}{\left(\sum_{i}S_{i}^{*}\right)}+\frac{1}{S}.$$

Though we recommend using expression 10 for practical applications, 11 provides some useful insight. By increasing the number of permutations, the contribution from the term on the left can be reduced, however, if it is already small relative to the term on the right, then the benefits of additional permutations will be minimal. Also, it becomes clear that when the total number of tests conducted is large relative to the number of positive tests, the variance in $\text{F}\hat{\text{D}}\text{R}$ is almost strictly a function of *positive* test counts and *not* dependent on the total number of tests conducted.

A confidence interval (CI) estimator for FDR can be developed in a manner analogous to the approach commonly used for the odds ratio that is, an exponential back-transform with a normal approximation,

(12) $${\text{CI}}_{\text{FDR}}=\exp\left\{\log\left(\text{F}\hat{\text{D}}\text{R}\right)\pm z_{\alpha /2}\sigma_{\text{FDR}}\right\}.$$

It is important to note that the variance and thus the CI is undefined when the number positive test results in the permuted data is zero. When this occurs we take the conservative approach of setting this number to one for estimation of the CI.

The development of the variance estimator relies on the assumption that the positive test counts follow a binomial distribution. Thus, tests are assumed to be i.i.d. Bernoulli variables. This assumption has two parts, (1) the tests are independent and (2) identically distributed that is, the probability of a positive result is the same for all tests.

The second property can be described as exchangeability across tests in the sense that each test is assumed to yield a positive outcome with the same probability *p*. In theorem 1 of Appendix B, “variance inequality of a binomial sum,” we show that a cryptic binomial mixture may cause an upward but not a downward bias in the variance estimate, implying that a departure from exchangeability across tests could cause the variance estimator to be more conservative but not more anti-conservative. We also found in simulations that the binomial variance estimator is highly robust to departures, and that in extreme cases where substantial departures do occur, the estimator does indeed become more conservative (data not shown).

On the other hand, the independence assumption (1) does present a major concern and is addressed here by modifying the variance estimator with an over-dispersion parameter to account for dependencies. This parameter can be estimated directly from counts of positive tests and thus does not require an additional analysis of the raw data or even the full set of test results. In contrast, Efron (2007a, 2010a) proposed a correction based on an estimator of root mean squared correlation in an underlying dataset. However, there is the requirement that dependencies among tests are represented by pairwise correlations between variables represented in a dataset, which is often not the case, e.g., eQTL analysis. Also, an additional analysis must be conducted using the primary data. Our approach is more general, does not require revisiting the primary data, and is more efficient in terms of data storage requirements because it uses positive test counts only.

### Over-dispersion estimator

In practice, most genomic datasets include dependencies between features that ultimately result in dependencies between tests, although the correspondence can be quite complex. For typical hypothesis tests that evaluate associations between molecular and phenotypic traits, positive or negative correlations between traits lead to positive correlations between tests causing over-dispersion in the variance of positive test counts (Edwards, 1960), which in turn causes over-dispersion in the variance of $\text{F}\hat{\text{D}}\text{R}$. We introduce an over-dispersion parameter to account for these dependencies.

The over-dispersion parameter is used to scale the variance estimate for $\text{log}(\text{F}\hat{\text{D}}\text{R})$ and is not needed (fixed at 1) if tests are known to be independent. Replicate positive test counts in the permuted data provide a convenient opportunity to assess dependence-induced over-dispersion without the necessity of revisiting the raw data or additional computationally expensive resampling procedures as proposed by Storey (2002) for FDR CI estimation. Each term in the expression for the variance of $\text{log}(\text{F}\hat{\text{D}}\text{R})$ includes a component factor, which is a variance estimate for positive test counts (Appendix B), thus an estimate of over-dispersion of positive test counts could be used as a scalar parameter for the variance of $\text{log}(\text{F}\hat{\text{D}}\text{R})$. The concept is to use permuted datasets to construct a ratio of the sample variance of positive test counts to the estimated variance based on the sample mean,

(13) $$\hat{\phi }=\frac{\left(\sum {\left(S_{i}^{*}-{\bar{S}}^{*}\right)}^{2}\right)/\left(B-1\right)}{m\hat{p}\left(1-\hat{p}\right)},\;\hat{p}=\frac{{\bar{S}}^{*}}{m},\;\sigma_{\text{FDR}\left(a\right)}^{2}=\hat{\phi }\sigma_{\text{FDR}}^{2}$$

where “*a*” indicates adjustment for dependencies.

## Data analysis

### Bias and variance of the proposed point estimator

We compared the proposed estimator with the ST and Efron (2010a) approaches to characterize differences in bias and variance over a range of conditions. Case-control data were simulated with dependences by fixing the root mean squared correlation at three levels according to the R function “simz” (Efron, 2010b). *Z*-scores were simulated for 100 cases and 100 controls at 2000 “genes” with false null hypotheses created by adding a constant to case observations, as described by Efron (2010b). The constant was fixed at 0.15 and 0.3 to reflect weak vs. strong effects, which yield differing numbers of false null hypotheses with test statistics below the detection threshold, *m*_1_ − *T* > 0. *P*-values were generated using *t*-tests, and for the ST and Efron (BE) estimators, they were adjusted using 10 or 100 permuted datasets.

**Figure 1:**
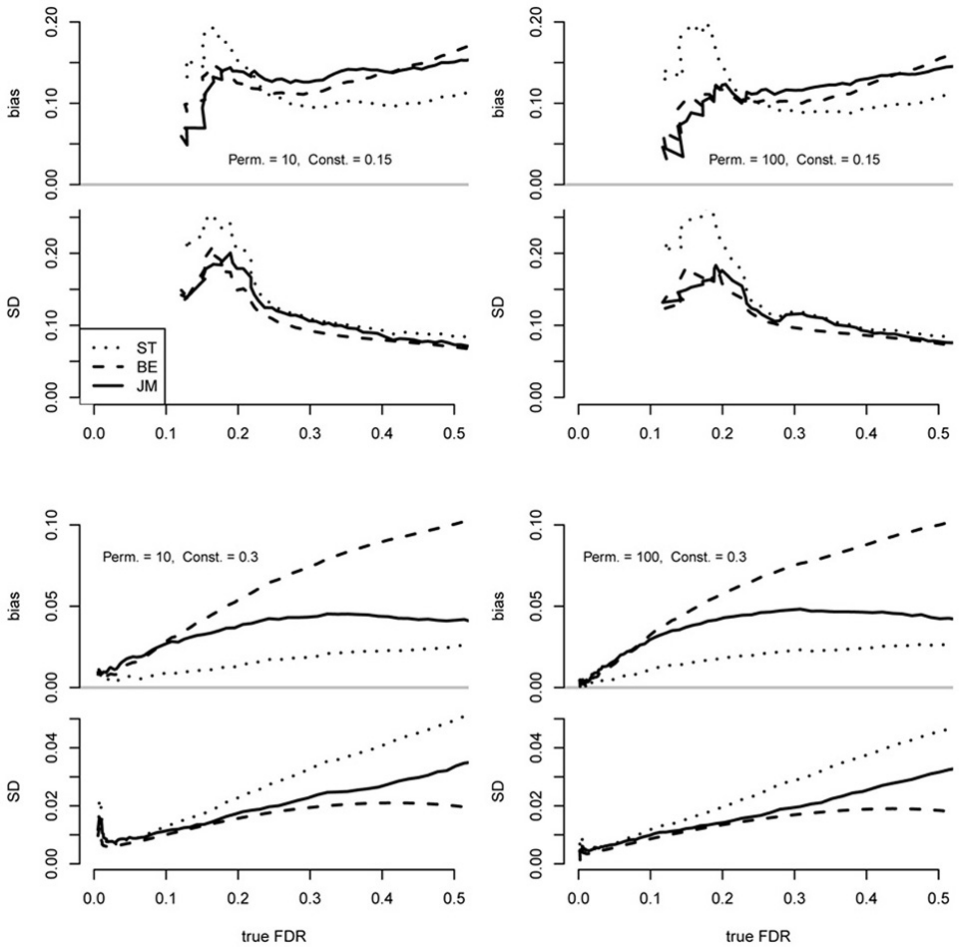
**Performance of the proposed FDR point estimator (JM; implemented in the “fdrci” R package) as compared to the Storey and Tibshirani approach (ST) as implemented in the “*q*-value” R package and the Efron approach (BE) as implemented in the “locfdr” R package**. Each plot was based on 200 replicate datasets independently simulated under identical conditions using the simz software (Efron, 2010a,b), where dependencies are determined by fixing the root mean squared correlation, denoted by α, of the raw data to 0.05. From each dataset, 2000 *t*-tests of 100 “cases” and 100 “controls” were generated, where false null hypotheses were defined by adding a constant to the raw simulated *z*-scores of “cases,” as described by Efron (2010b) and π_0_ = 0.75. Data were simulated with 40 blocks of correlated *z*-scores according to α. Case-control labels were randomly permuted 10 or 100 times (*B*) for each scenario. Differing values of “true FDR” reflected a series of increasing significance thresholds. True FDR was computed from the simulated data as mean *F*/*S*. Bias was computed as the mean $\text{F}\hat{\text{D}}\text{R}$—true FDR.

As expected, all methods were conservatively biased in all scenarios across a range of significance thresholds (Figure 1). Also, results were very similar overall between 10 and 100 permutations (*B*), implying that under these conditions little improvement is achieved by the order-of-magnitude increase in *B*. This result is consistent with Equation 11 that shows a small contribution in the variance due to permutations when the number of positive tests in permuted data is substantial.

When the effects were weak (constant = 0.15) the ST estimator was more conservatively biased than the others between approximately FDR = 0.1–0.2, and this divergence increased with the increased number of permutations (Figure 1). Also, variance of the ST was greater over this range. However, it was less biased than the proposed (JM) and BE estimators above this range while maintaining a similar variance. The JM and BE performed similarly under these conditions with neither out-performing the other in bias or variance across the entire range.

In contrast, when the effects were stronger (constant = 0.30), the ST was less biased than the others across the entire range but the variance was greater over most of the range. This bias-variance tradeoff is also apparent in the difference between the JM and EB estimators with the JM substantially less biased over the approximate range FDR > 0.1 but with greater variance. From FDR = 0–0.1, JM and BE performed quite similarly, but ST bias was smaller and the variance was comparable.

### Performance of FDR variance and CI estimators

**Figure 2:**
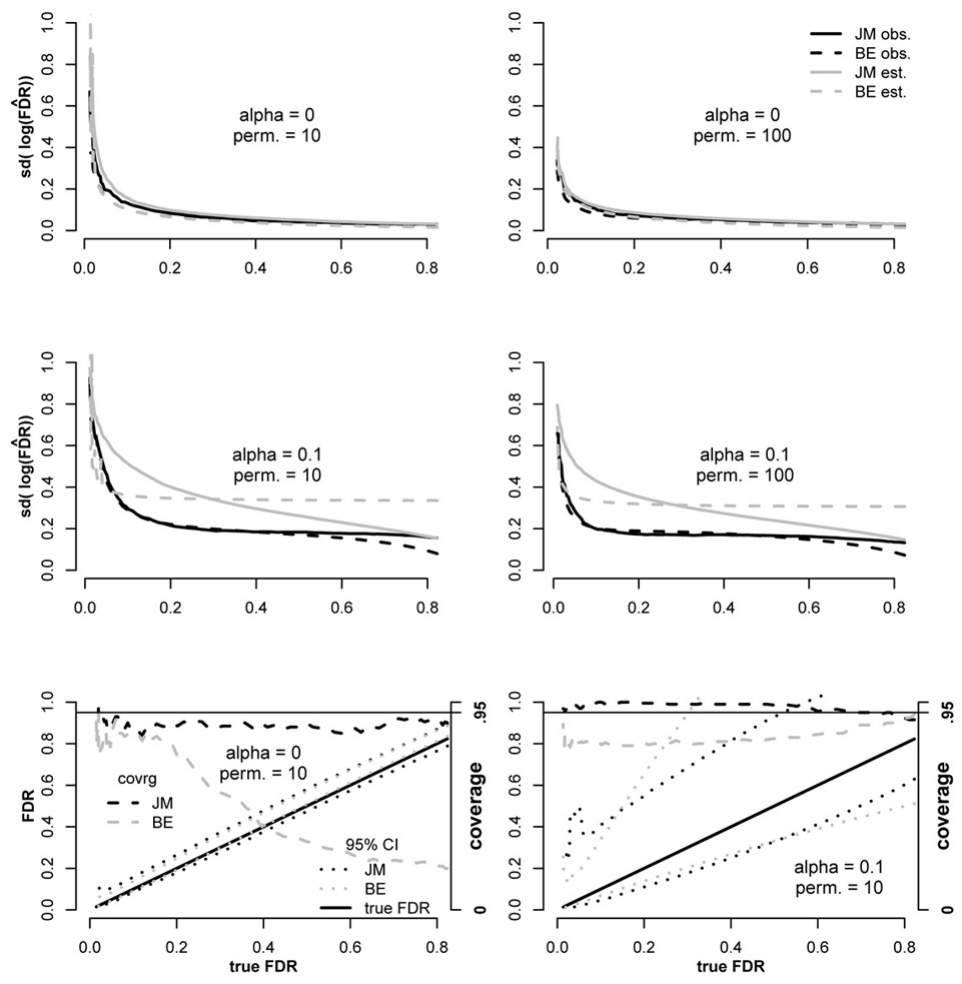
**Comparison of the Bradley Efron (2010a,b) estimator for variance of log(FDR) to the proposed estimator (JM) under independence among tests (α = **0**) and dependence (α = **0.1**)**. Coverage of derived 95% CI's were also compared in the bottom plots according to Equation 12.

We compared our proposed variance estimator for $\text{log}(\text{F}\hat{\text{D}}\text{R})$ to the estimator proposed by Efron (2010a) both under independence between tests and when dependencies were present (Figure 2). Simulations were performed as described above except that 4000 “genes” were tested for each replicate, 400 of which corresponded to false null hypotheses, with constant = 0.3.

From Figure 2 it is clear that when tests were independent (α = 0), estimates for both estimators were close to observed values both for 10 and 100 permutations. However, when dependencies were simulated (α = 0.1), both methods were conservatively biased over most of the range. Below FDR ≈ 0.3 the JM estimator was more conservative than the BE and above 0.3 it was less conservative. The EB estimator was anti-conservative for FDR < 0.07 when 10 permutations were conducted but not when the number of permutations was increased to 100.

Using the BE variance estimator, we constructed CIs as proposed in Equation 12 to compare this approach to the proposed JM CI estimator. The JM 95 percent CI estimator outperformed the BE estimator in both the independent and dependent testing scenarios (Figure 2). The poor coverage of the BE estimator under independence is mostly due to upward bias that results in the lower bound exceeding the true FDR. Coverage of the JM estimator is slightly below the 95 percent target for the same reason, an upward bias. It's important to note that exact coverage is not as important when the CI width is small, as is the case in the independent scenario. The coverage problem for the BE estimator is not as severe in the dependent testing scenario, however, it is still well-below 95% and the mean CI width is substantially larger than the proposed estimator over most of the range. The coverage of the JM CI estimator is better than that of the BE estimator in the dependent scenario as well, meeting or exceeding 95% over most of the domain even though the mean JM width tends to be smaller.

**Figure 3:**
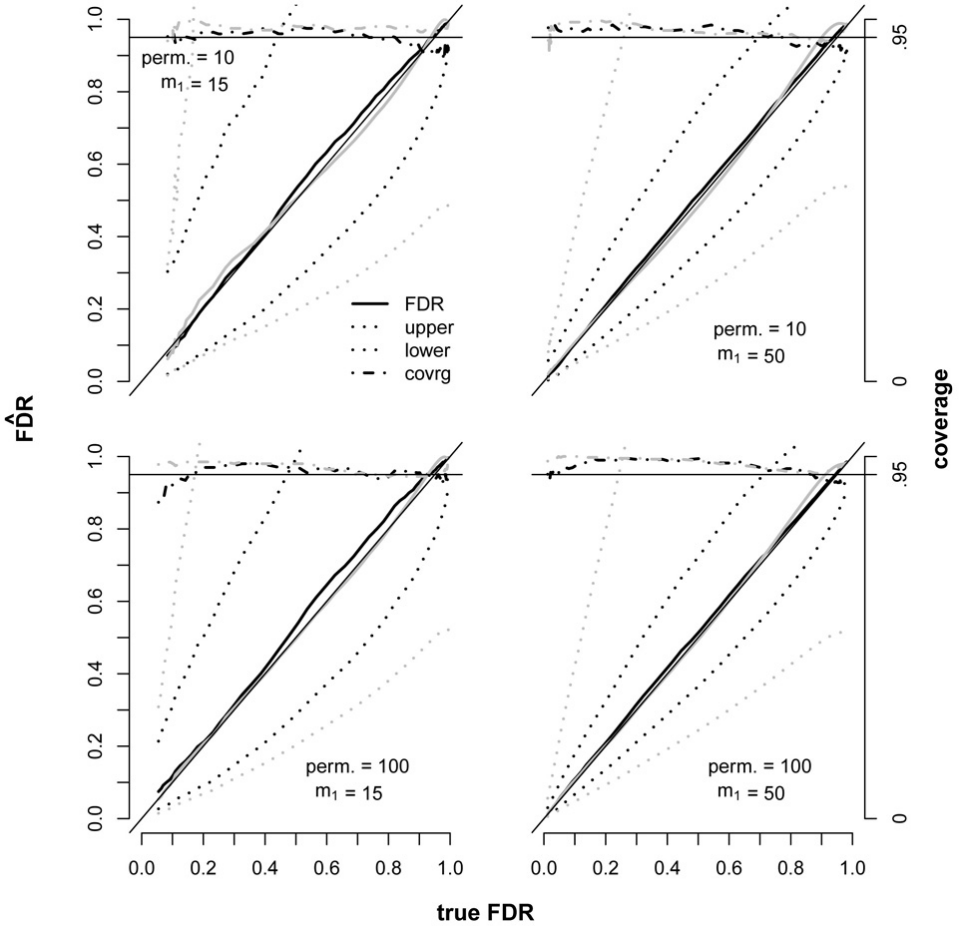
**Performance of the JM (black) and BE (gray) 95% CI estimators in the presence of dependent tests**. Each plot represents 200 replicate datasets independently simulated under identical conditions. The true fdr ranged along the x-axis due to applying a variety of significance thresholds. Each dataset corresponded to 5050 tests. The number of false null hypotheses (*m*_1_) was fixed at either 15 or 50. The thin solid black line along the diagonal represents unbiasedness and the thicker solid lines denote FDR point estimates. Means for upper and lower 95 percent confidence bounds are shown as dotted lines. The target confidence interval coverage of.95 is displayed as a solid horizontal line at 0.95 and actual coverage by dashed lines. SNPs were generated in “LD blocks” with 5 SNPs per block and composite LD ranging from 0.4 to 0.9 within each block, and traits were generated in “modules” of correlated traits with 5 traits per module and correlations ranging from 0.4 to 0.9 within each module. Twenty LD blocks and 10 gene modules were included in each replicate dataset.

To explore the performance of the methods under a different set of realistic genomic testing conditions, SNPs and Gaussian traits were simulated with dependencies and then tested for associations using linear additive models. The HAPSIM (Montana, 2005) R package was used to randomly generate haplotypes corresponding to specified ranges of LD, from which the SNP data was constructed. Allele frequencies were sampled from a uniform (0.2, 0.5) distribution. Data were simulated under two different proportions of false null hypotheses, each employing 10 and 100 permutations (Figure 3). For each of these four scenarios, CI's were computed using the JM and BE variance estimators under a range of significance thresholds. This study scenario presented a challenge for the BE approach because there were two datasets used for testing (SNPs and Gaussian traits), both with dependencies. In contrast, the guidance given by Efron (Efron, 2010a,b) dealt with just a single underlying dataset of correlated variables yielding a one-to-one mapping from variables to tests. In lieu of a formal method to compute an overall alpha (mean squared correlation) for the multiple dataset scenario (required by the BE method to adjust for dependencies) we used the mean alpha across datasets. In contrast, no alteration of the JM approach was necessary, since the over-dispersion parameter is computed strictly from positive test counts.

Biases of the point estimators were small and the JM estimator was slightly conservative where the bias was noticeable, as expected (Figure 3). Coverages of the CI estimators were generally conservative as well, hence the proposed over-dispersion parameter demonstrated an adequate ability to correct for dependencies. However, mean widths of the BE CI's were extremely wide compared to the JM widths, implying that the heuristic approach of taking the mean alpha across datasets was not adequate. This problem highlights the sensitivity of the BE variance estimator to the type of data and tests conducted due to the computation of alpha, and in this case an appropriate method has not yet been described.

There was one small region where coverage of the JM CI was slightly low. The low coverage occurred where FDR was small, the number of false null hypotheses was small (15), and the number of permutations was 100 (bottom left panel of Figure 3). The somewhat low coverage in this region can be explained by the conservative bias of the point estimator combined with small CI widths, thus it is unlikely to be a problem in practice. When the number of false null hypotheses was increased to 50, coverage was more conservative and no longer low over this region. In general, increasing the number of false null hypotheses had a substantial decreasing effect on CI widths, as implied by Equation 11, but the effect of increasing the number of permutations from 10 to 100 was very modest. It is important that FDR CI coverage is good in the case where all null hypotheses are true, and we found that coverage of the JM estimator was conservative under these conditions (data not shown).

### Mouse gene expression in hypothalamus is predictive of REM sleep

We investigated the relationship between rapid eye movement (REM) sleep and transcriptome-wide gene expression variation in male mice from a genetically segregating back-cross population of inbred mouse lines, C57BL/6J and BALB/cByJ, both the breeding scheme and sleep measures described previously (Winrow et al., 2009). These datasets were downloaded from a public database hosted by Sage Bionetworks (www.synapse.org; dataset IDs for the sleep phenotypes and hypothalamus gene expression were syn113322 and syn113318, respectively). One hundred and one mice were hand scored for sleep at 11–13 weeks of age using electroencephalogram (EEG) and electromyogram (EMG) data collected over a 48 h period (Winrow et al., 2009; Brunner et al., 2011; Millstein et al., 2011; Fitzpatrick et al., 2012). Hypothalamus tissue was collected from each mouse and profiled following sleep recording (Millstein et al., 2011) to identify chronic gene expression variation associated with variation in 24 h REM sleep. After an extensive quality control process applied to the gene expression data that included removal of probes containing SNPs and probes that were not considered to be poly-A reliable, a total of 17,404 probes remained for analysis.

For all 17,404 probes, *F*-tests of coefficients from linear models were used to test for associations between gene expression and mean 24 h REM sleep across the 48 h recording period, where both gene expression and REM sleep duration were coded as continuous variables with a single observation per animal. None of the resulting *p*-values achieved a typical Bonferroni significance level for family-wide α = 0.05 (*p* < 2.87e-6) or even a BH FDR equal 0.05 significance level. There is very little guidance in the literature regarding what to do when this happens, publish a negative finding? The problem here is that although there may be some evidence in the data of a true biological signal that signal may be too weak to achieve a Bonferroni or BH 0.05 significance level. However, using the proposed FDR CIs, the investigator is able to relax the significance threshold if necessary to capture and quantify evidence for relatively weak biological signals.

**Figure 4:**
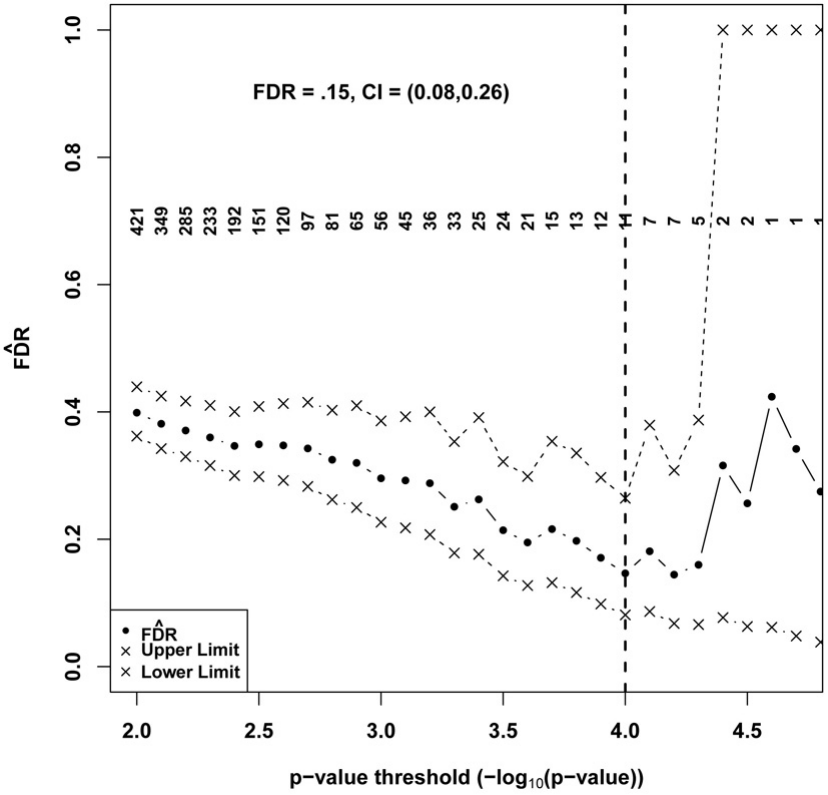
**Estimated FDR and 95% CI for a series of significance thresholds applied to 17,404 tests of association between gene expression features and 24 h REM sleep**. A final set of “significant” genes was identified using a threshold, shown as a vertical black dashed line that corresponded to the minimum $\text{F}\hat{\text{D}}\text{R}$ and minimum upper confidence limit. Numbers in the field denote counts of positive tests at the specified *p*-value significance threshold.

Figure 4 shows $\text{F}\hat{\text{D}}\text{R}$ generated according to the proposed method plotted with CIs based on 1000 permutations over a range of potential *p*-value significance thresholds. Each permuted dataset was created by randomly permuting the individual labels corresponding to expression data. This approach preserves observed dependencies between transcripts. Ultimately, an investigator often choses a single “significance” threshold (typically a Bonferroni adjusted.05 alpha level) and reports those findings that meet the criterion, considering these to be “discoveries” that are worth further investigation. Unlike FWER control, where a universal threshold such as.05 can function as a single interpretable criterion to define significant features and quantify uncertainties, applying a FDR estimation approach may yield a range of thresholds over which $\text{F}\hat{\text{D}}\text{R}$ is significantly less than one but the number of discoveries and the magnitude of $\text{F}\hat{\text{D}}\text{R}$ varies. There is a trade-off between the number of true discoveries and the FDR, and the final choice should reflect the objectives of the study and the costs vs. benefits of false vs. true discoveries. In these results, a minimum $\text{F}\hat{\text{D}}\text{R}$ and minimum upper confidence limit coincided approximately to define a natural threshold at *p* < 0.0001 $\left[\text{F}\hat{\text{D}}\text{R}=0.15\;(0.08,\;0.26)\right]$, yielding 11 transcripts. At this FDR level we would expect roughly 2 of the 11 to be false discoveries. Using this threshold, the BH method also determines FDR to be 0.15, suggesting that the parametric assumptions of the test are likely to be justified in this application. It is interesting to note that a consequence of choosing a minimum $\text{F}\hat{\text{D}}\text{R}$ is that among tests that achieve the chosen significance threshold, there is no evidence that smaller *p*-values are more likely to be true discoveries. In view of the small differences in $\text{F}\hat{\text{D}}\text{R}$ demonstrated above between 10 and 100 permutations, we did not believe that additional permutations would substantially improve our estimate or affect our ultimate choice of a significance threshold.

Though the 11 identified transcripts (supplementary Table S1) do not include genes well-known to regulate sleep, what is known about these genes does include some plausible links. For example, the two genes with the smallest *p*-values are secreted Frizzled-related proteins, Sfrp1 and Sfrp4 (*p* = 1.1e-5 and 3.1e-5, respectively), known to be involved in wnt signaling (Bovolenta et al., 2008) as well as dopamine neuron development (Kele et al., 2012). Wnt signaling has been linked to pathologies, mood and mental disorders, as well as neurodegenerative disease (Oliva et al., 2013), all of which commonly include sleep indications as comorbidities. Also, Irf7 and Ifit1 are involved in interferon signaling, a process found to affect both REM and non-REM sleep (Bohnet et al., 2004). Iigp2, a member of the p47 GTPase family, may also play a role in interferon signaling (Miyairi et al., 2007). Interferon induced with helicase C domain 1 (Ifih1) is upregulated in response to beta-interferon, and genetic variation in this gene has been found to be associated with type 1 diabetes (Winkler et al., 2011), which includes sleep disturbances as part of the long-term syndrome (Van Dijk et al., 2011).

## Discussion

The proposed method provides an accessible and computationally efficient approach for FDR CI estimation that accounts for dependencies among tests and the number of permutations conducted. Thus, it can easily be applied to genomic data, where dependencies are pervasive and the number of permutations often limited by computational resources. The method presents a major advance in addressing the oft-asked question, “how many permutations are required?” Even if a small number of permutations have been conducted, the investigator can be confident that this source of variance is reflected in the CI estimation, thereby adequately quantifying uncertainty in the FDR. The ability to apply this approach using only counts of tests that meet some threshold of interest is an important advantage that allows the method to be easily applied in very high dimensional testing settings such as trans eQTL, where storage of all test results or an additional analysis of raw data would be a computational burden. Also, the approach can be applied directly to statistics with uncharacterized distributions, bypassing the need for *p*-values entirely. Thus, there is no assumption of uniform or unbiased *p*-values. The main assumption is that permuted results accurately reflect the null.

The appropriateness of parametric distributions becomes a much more challenging issue in large-scale inference settings because the investigator is forced to work in the extreme tails to adjust for multiplicity. This problem is sometimes addressed by severe transformations such as quantile normalization (Becker et al., 2012), which can cause a loss in power due to a loss of information. The use of permutations in the proposed approach provides a flexible as well as powerful multiple-testing approach, which does not require loss-of-information transformations. Also, without permutations, it would be necessary to go back to raw data to account for dependencies in the quantification of FDR uncertainty. Thus, the method is useful even when all parametric assumptions are completely justified.

Simulation analysis demonstrated that variance of FDR estimators increased when there were dependencies between tests, in agreement with Schwartzman and Lin (2011). However, the proposed over-dispersion parameter adequately adjusted the CI under the conditions explored to account for this inflation. We showed both theoretically and via simulations that variance of the proposed FDR point estimator was more sensitive to the numbers of positive tests than the numbers of permutations. Indeed, there was little change in variance from 10 to 100 permutations. The proposed point estimator performed well, showing moderate and stable characteristics with regard to the bias-variance trade-off, out-performing the BE method in bias and the ST method in variance.

Both the proposed and BE estimators for $\text{log}(\text{F}\hat{\text{D}}\text{R})$ performed well when tests were independent but conservatively when dependencies were present (the anti-conservative behavior of the BE estimator was not present when permutations were increased to 100). Coverage of the proposed CI was mostly conservative, and it almost uniformly out-performed the CI constructed from the BE estimators.

We showed that the precision of the proposed point estimator depends primarily on the number of positive tests (and dependencies among tests), which is not directly related to the magnitude of $\text{F}\hat{\text{D}}\text{R}$. The ability to estimate a CI for FDR allows the investigator to identify sets of positive tests that are highly enriched for true positives yet are characterized by what would often be considered unreasonably high $\text{F}\hat{\text{D}}\text{R}$, such as 0.2 and above. Undoubtedly, there are many such datasets with true biological signals that have gone unpublished due to an inability to achieve statistical significance with conventional FWER or FDR thresholds. Conversely, results may have been published that were not justified by the strength of the evidence. The proposed CI estimator thus allows decoupling of “statistical significance” from the magnitude of the FDR estimate. However, caution should be used in treating the CI as a hypothesis test for determining whether FDR is statistically significantly smaller than one. When an investigator uses a *post-hoc* strategy for identifying the significance threshold (such as the threshold that yields the minimum $\text{F}\hat{\text{D}}\text{R}$ or minimum upper CI bound), the upper CI bound should be substantially below one to conclude that FDR is statistically significantly below one. Based on our experience in simulated data and permuted real data (data not shown), we suggest a rule-of-thumb defined by an upper bound below 0.7 where there are at least 5 positive tests at the chosen significance threshold (smaller upper bound if there are fewer) is likely to be sufficiently conservative for most situations. However, a thorough treatment of this important question is beyond the scope of this report. We leave it to future studies to elucidate just how this criterion depends on factors such as the number of permutations, the number of positive tests, and dependencies among tests.

Not only were suggestive links found in the literature between REM sleep and gene expression for the set of 11 genes whose expression was significantly associated with 24 h REM sleep, but the signal-to-noise ratio was also quantified in the form of FDR, along with a measure of uncertainty in the estimate. From the sleep data analysis, it is clear that there is evidence of association between gene expression and REM sleep, and we are able to identify many of the genes likely to be involved. If a typical FWER approach or a BH FDR approach had been applied to these data, the investigator would have failed to reject the global null hypothesis of no association between gene expression and REM sleep. Though 11 genes may seem like a small number, it is important to remember that these associations reflect chronic differences in expression and sleep between individuals (all individuals were sacrificed at the same point in the light/dark cycle) as distinct from detecting genes that cycle with sleep state changes. Also, we set out to identify genes that explain normal sleep variation in individuals who are relatively healthy, unlike many differential expression studies that are conducted by comparing a diseased or perturbed population, e.g., sleep deprivation, to a healthy one.

The migration to non-parametric approaches in genomic analyses may be inevitable as investigators are faced with seemingly insurmountable challenges of satisfying parametric assumptions in the context of many thousands of sample distributions. In addition, the typically stringent significance thresholds used in multiple testing on a genomic scale results in the need to draw inferences based on the extreme tails of an assumed distribution, which are notoriously inaccurate. Permutation-based approaches are attractive in their flexibility and accuracy but are computationally expensive. We have described a method (with software freely available as an R package, “fdrci”: http://cran.r-project.org/web/packages/fdrci/index.html) where permutations can be used to estimate FDR including CIs in a fully non-parametric approach, which is computationally parsimonious and robust to dependencies among tests.

### Conflict of interest statement

The authors declare that the research was conducted in the absence of any commercial or financial relationships that could be construed as a potential conflict of interest.

## Appendix A

### FDR consistency

If we assume that individual hypothesis tests are consistent, then as sample size, *n*, goes to infinity, power of each individual test goes to 1, therefore,

$$\left(m_{1}-T\right)\overset{n\;\to \;\infty }{\to }0\Rightarrow (m-S)\overset{n\;\to \;\infty }{\to }\left(m_{0}-F\right)$$

By design, the permuted dataset should accurately represent a realization from the complete null. If this is the case, then,

$$\frac{E\left[{\bar{S}}^{*}\right]}{m}=\frac{E[F]}{m_{0}},$$

and assuming that π_0_ is fixed,

$$\frac{{\bar{S}}^{*}}{m}\overset{m\;\to \;\infty }{\to }\frac{F}{m_{0}}.$$

Due to binomial properties, the variances of the above proportions go to zero as *m* goes to infinity. Therefore, as *m* and *n* go to infinity,

$$\begin{array}{l} {\hat{\pi }}_{0}=\frac{1-S/m}{1-{\bar{S}}^{*}/m}=\frac{m-S}{m(1-F/m_{0})}=\frac{m_{0}-F}{m-Fm/m_{0}}\\ \;=\frac{m_{0}\left(1-F/m_{0}\right)}{m-Fm/m_{0}}=\frac{m_{0}\left(m-Fm/m_{0}\right)}{m\left(m-Fm/m_{0}\right)}=\frac{m_{0}}{m}. \end{array}$$

Thus ${\hat{\pi }}_{0}$ is a consistent estimator in *m* and *n*. Even if *m* does not go to infinity, the above shows that bias in ${\hat{\pi }}_{0}$ will go to zero as *n* goes to infinity.

## Appendix B

### Variance of S

The development of a variance estimator for $\text{log}(\text{F}\hat{\text{D}}\text{R})$ depends on an estimator for the variance of *S*. We use the approximation that *S* is a binomial random variable, which has an obvious rational under the global null but is more complicated under the alternative, where *T* > 0. In this case *S* can be thought of as a sum of two binomial variables, *F* ~ Bin(*m*_0_, *E*[*F*]/*m*_0_) and *T* ~ Bin(*m*_1_, *E*[*T*]*/*m*_1_*), where the sum, *S* = *F* + *T*, is not necessarily binomially distributed. However, the proposed binomial variance approximation will be a conservative estimator.

### Theorem 1

#### Variance inequality of a binomial sum

Suppose the sum, *Z*, of two independent binomial random variables, *X* ~ *B*(*m*_0_, *p*_0_) and *Y* ~ *B*(*m*_1_, *p*_1_), *Z* = *X* + *Y*. Then the variance of *Z* is less then or equal to its variance under a binomial distribution that is, Var(*Z*) <= *E*[*Z*] (1 − *E*[*Z*]/(*m*_0_ + *m*_1_)).

Proof. The random variables *X* and *Y* are independent; therefore the variance of the sum is the sum of the variances, *Var*(*Z*) = *E*[*X*](1 − *E*[*X*]/*m*_0_) + *E*[*Y*](1 − *E*[*Y*]/*m*_1_). Thus, we need to show that, *E*[*X*](1 − *E*[*X*]/*m*_0_) + *E*[*Y*](1 − *E*[*Y*]/*m*_1_) ≤ *E*[*Z*](1 − *E*[*Z*]/(*m*_1_ + *m*_0_)). Simplifying this inequality yields,

$$\begin{array}{l} E[X](1-E[X]/m_{0})+E[Y](1-E[Y]/m_{1})\leq (E[X]+E[Y])(1-(E[X]+E[Y])/(m_{0}+m_{1}))\\ E[X]-E{\left[X\right]}^{2}/m_{0}+E[Y]-E{\left[Y\right]}^{2}/m_{1}\leq E[X]+E[Y]-{\left(E[X]+E[Y]\right)}^{2}/(m_{0}+m_{1})\\ E{\left[X\right]}^{2}/m_{0}+E{\left[Y\right]}^{2}/m_{1}\geq {\left(E[X]+E[Y]\right)}^{2}/(m_{0}+m_{1})\\ \frac{m_{1}(m_{0}+m_{1})E{\left[X\right]}^{2}}{m_{0}m_{1}(m_{0}+m_{1})}+\frac{m_{0}(m_{0}+m_{1})E{\left[Y\right]}^{2}}{m_{0}m_{1}(m_{0}+m_{1})}\geq \frac{m_{0}m_{1}{\left(E[X]+E[Y]\right)}^{2}}{m_{0}m_{1}(m_{0}+m_{1})}\\ m_{1}(m_{0}+m_{1})E{\left[X\right]}^{2}+m_{0}(m_{0}+m_{1})E{\left[Y\right]}^{2}\geq m_{0}m_{1}(E{\left[X\right]}^{2}+2E[X]E[Y]+E{\left[Y\right]}^{2})\\ m_{1}^{2}E{\left[X\right]}^{2}+m_{0}^{2}E{\left[Y\right]}^{2}+m_{0}m_{1}E{\left[X\right]}^{2}+m_{0}m_{1}E{\left[Y\right]}^{2}\geq m_{0}m_{1}E{\left[X\right]}^{2}+2m_{0}m_{1}E[X]E[Y]+m_{0}m_{1}E{\left[Y\right]}^{2}\\ m_{1}^{2}E{\left[X\right]}^{2}+m_{0}^{2}E{\left[Y\right]}^{2}\geq 2m_{0}m_{1}E[X]E[Y]\\ m_{1}^{2}E{\left[X\right]}^{2}-2m_{0}m_{1}E[X]E[Y]+m_{0}^{2}E{\left[Y\right]}^{2}\geq 0\\ {\left(m_{1}E[X]-m_{0}E[Y]\right)}^{2}\geq 0 \end{array}$$

which clearly is true for all independent binomial distributions of *X* and *Y*. Though theorem 1 was developed for the sum of two variables, it easily generalizes to *k* > 2.

## Appendix C

### Variance of $\text{log}(\text{F}\hat{\text{D}}\text{R})$

The variance of the log FDR estimate can be described as the variance of the sum of two independent quantities that is,

$$\begin{array}{l} \text{Var}\left(\log\left(\text{F}\hat{\text{D}}\text{R}\right)\right)=\text{Var}\left(\log\left(\frac{{\bar{S}}^{*}}{1-{\bar{S}}^{*}/m}\times \frac{1-S/m}{S}\right)\right)\\ \;=\text{Var}\left(\log\left(\frac{(1/B)\sum S_{i}^{*}}{1-\sum S_{i}^{*}/mB}\times \frac{1-S/m}{S}\right)\right)\\ \;=\text{Var}\left(\log\left(\frac{\sum S_{i}^{*}}{mB-\sum S_{i}^{*}}\right)\right)+\text{Var}\left(\log\left(\frac{m-S}{S}\right)\right) \end{array}$$

thus due to independence between *S* and *S*^*^, the variance of the sum is the sum of the variances.

Using the Delta method and the normal approximation to the binomial, we know that each term and the sum of terms converge to a normal distribution. It is true that *S* is actually a mixture distribution from true and false null hypotheses, but to the extent that this fact biases the variance, it will be a conservative bias. This follows from theorem 1 (above) and the resulting expression from the Taylor approximations (below).

With a first order Taylor approximation, we can approximate the variance of the first term as,

$$\text{Var}\left(\log\left(\frac{\sum S_{i}^{*}}{mB-\sum S_{i}^{*}}\right)\right)\approx \left[g^{\prime }\left(\sum S_{i}^{*}\right)\right]\times \text{Var}\left(\sum S_{i}^{*}\right),$$

where

$$g^{\prime }\left(\sum S_{i}^{*}\right)=\frac{\partial }{\partial \sum S_{i}^{*}}\log\left(\frac{\sum S_{i}^{*}}{mB-\sum S_{i}^{*}}\right).$$

By the chain rule,

$$\frac{\partial }{\partial \sum S_{i}^{*}}\frac{\sum S_{i}^{*}}{mB-\sum S_{i}^{*}}=\frac{mB}{{\left(mB-\sum S_{i}^{*}\right)}^{2}}$$

and

$$\begin{array}{l} g^{\prime }\left(\sum S_{i}^{*}\right)=\frac{mB-\sum S_{i}^{*}}{\sum S_{i}^{*}}\times \frac{mB}{{\left(mB-\sum S_{i}^{*}\right)}^{2}}\\ \;=\frac{mB}{\sum S_{i}^{*}\left(mB-\sum S_{i}^{*}\right)}. \end{array}$$

Thus, for the first term,

$$\text{Var}\left(\log\left(\frac{\sum S_{i}^{*}}{mB-\sum S_{i}^{*}}\right)\right)={\left(\frac{mB}{\sum S_{i}^{*}\left(mB-\sum S_{i}^{*}\right)}\right)}^{2}\times \sum S_{i}^{*}\left(1-\frac{\sum S_{i}^{*}}{mB}\right)=\frac{mB}{\sum S_{i}^{*}\left(mB-\sum S_{i}^{*}\right)}.$$

Taking a similar approach for the second term, $\frac{\partial }{\partial S}\log\left(\frac{m-S}{S}\right)=\frac{S}{m-S}\times \frac{-m}{S^{2}}=\frac{m}{S(S-m)}$, and with a first order Taylor approximation, $\text{Var}\left(\log\left(\frac{m-S}{S}\right)\right)\approx \left[g^{\prime }\left(\sum S_{i}^{*}\right)\right]\times \text{Var}\left(\sum S_{i}^{*}\right)=\frac{m}{S(m-S)}$. In summary, the variance of the log of is the sum of variances, $\text{Var}\left(\log\left(\text{F}\hat{\text{D}}\text{R}\right)\right)={\hat{\sigma }}_{\text{FDR}}^{2}=\frac{mB}{\sum S_{i}^{*}\left(mB-\sum S_{i}^{*}\right)}+\frac{m}{S\left(m-S\right)}$.

The same result can be arrived at by conceptualizing the FDR estimate as an odds ratio between results in observed vs. permuted data. That is, we can construct the following 2 × 2 table:

**Table:**

		Positive test?		
		Yes	No	
Permuted data?	Yes	∑ *S*^*^_*i*_	*mB* − ∑ *S*^*^_*i*_	*mB*
	No	*S*	*m* − *S*	*m*

Seen in this context, it is clear that the proposed FDR point estimator takes the simple form of the odds ratio of test results in the observed and permuted data. It is then also clear that the proposed variance expression, which can be written,

$$\sigma_{\text{FDR}}^{2}=\frac{1}{\left(\sum_{i}S_{i}^{*}\right)}+\frac{1}{mB-\sum_{i}S_{i}^{*}}+\frac{1}{S}+\frac{1}{m-S},$$

takes the form of the well-known expression proposed by Woolf (1955) for variance of the log odds ratio.
